# Perfusion imaging with 320-slice spiral computed tomography and color-coded digital subtraction angiography for assessing acute skeletal muscle ischemia-reperfusion injury in a rabbit model

**DOI:** 10.1186/s12880-019-0353-1

**Published:** 2019-08-28

**Authors:** Chengzhi Li, Xingyi Liu, Huimin You, Hong Zhang, Yuqi Liu, Yan Zhang

**Affiliations:** 1 Department of Interventional Radiology and Vascular Surgery, The First Affiliated Hospital of Jinan University, No.613 of West Huangpu Avenue, Guangzhou, 510630 China; 2 Graduate School, Guangzhou University of Chinese Medicine, Guangzhou, China; 3 Second work unit: Department of Interventional Radiology and Vascular Surgery, The First Affiliated Hospital of Jinan University, No.613 of West Huangpu Avenue, Guangzhou, 510630 China; 4 Department of endocrinology, The Fifth Affiliated Hospital of Guangzhou medical university, Guangzhou, 510700 China

**Keywords:** Ischemia-reperfusion injury, CT perfusion imaging, Color-coded DSA, Skeletal muscle

## Abstract

**Background:**

In recent years, skeletal muscle-related ischemia-reperfusion injury (IRI) has become more common. IRI can lead to severe limb injuries, multiple organ failure, and even death in some cases. However, there is still a lack of rapid and sensitive detection methods for IRI in skeletal muscle. This study aims to explore the value of computed tomography perfusion imaging (CTPI) and color-coded digital subtraction angiography (DSA) in assessing acute IRI of skeletal muscle in a rabbit model.

**Methods:**

Fifty New Zealand white rabbits were randomly assigned to the ischemia-reperfusion (IR) group (*n* = 40) or sham group (*n* = 10). After 3 h of surgically-induced hindlimb ischemia, the IR group underwent reperfusion and CTPI and color-coded DSA were taken to assess the skeletal muscle at 0, 6, 12, or 24 h post-reperfusion. The data from CTPI and DSA in the right and left hindlimbs, blood flow (AF-R/L), blood volume (BV-R/L), contrast clearance rate (C-R/L) and the maximum contrast enhancement values (peak-R/L) were obtained. Serum superoxide dismutase (SOD), creatine kinase (CK), lactic dehydrogenase (LDH) and malondialdehyde (MDA) were measured. The statistical correlation between the above parameters (CTPI, color-coded DSA, and biochemical markers) was analyzed.

**Results:**

The mean value of AF-R/L, BV-R/L, C-R/L and peak-R/L decreased linearly from 1.07 ± 0.08 to 0.75 ± 0.11, 1.03 ± 0.06 to 0.85 ± 0.14, 0.93 ± 0.15 to 0.71 ± 0.18, and 1.07 ± 0.01 to 0.47 ± 0.04, respectively. The correlation coefficients between AF-R/L and SOD, CK, LDH and MDA were 0.57, − 0.44, − 0.60, and − 0.62, respectively (*p* < 0.001). The correlation coefficients between Peak-R/L and SOD, CK, LDH, MDA were 0.59, 0.68, 0.71 and 0.66, respectively (*p* < 0.001). The correlation coefficient between AF-R/L and Peak-R/L was 0.70 (*p* < 0.001).

**Conclusion:**

Both CTPI and color-coded DSA could dynamically assess skeletal muscle IRI in rabbits.

## Background

Ischemia-reperfusion injury (IRI) is tissue damage that occurs when the blood supply is restored to tissues after a period of ischemia [1]. The restoration of the blood flow to these tissues leads to microvascular injury, oxidative stress, and inflammation [2]. In recent years, IRI involving skeletal muscle has become more common and can lead to severe limb injuries, multiple organ failure, and even death in some cases [3].

IRI can be evaluated by its clinical manifestations, including clinical symptoms, medical imaging, and measurement of certain serum biochemical biomarkers. Currently, the primary biomarkers evaluated in cases of IRI include creatine kinase (CK), lactate dehydrogenase (LDH), malondialdehyde (MDA), and hydrogen peroxide dismutase (SOD) [4]. While providing vital insight into disease severity, the measurement of these biomarkers requires time and may not be feasible during urgent traumas. For this reason, medical imaging had become a key player in the diagnosis and treatment planning of IRI conditions.

In terms of medical imaging, computed tomography perfusion imaging (CTPI), magnetic resonance imaging (MRI), and digital subtraction angiography (DSA) have been widely used for evaluating IRI in myocardium and brain tissue [5–8]. CTPI is one of the most widely used techniques for detecting and evaluating perfusion in tissues or organs [9]. The advantages of CTPI include its widespread availability, short acquisition times, and simple calculation for perfusion quantification. However, the high radiation exposure has limited the development of CTPI in some cases [10, 11]. CTPI is used to dynamically scan preselected regions of interest (ROI) within tissues or organs after injection of a contrast agent. After scanning, the time-density curve (TDC) of each pixel in the ROI is obtained. Based on the TDC, blood flow (BF), blood volume (BV), mean moving time of the contrast agent (MMT), and capillary permeability can be calculated using certain mathematical models, thus providing insight into the perfusion state [12].

Color-coded DSA, which is a post-processing technology used to analyze image density changes caused injected contrast agents, converts scan information into continuous-change pixels. The creation of a time-density curve of the bolus injection of the contrast agent in the user-defined regions allows for the calculation of functional parameters like blood-flow distribution and blood-flow velocity [13, 14].

CTPI and color-coded DSA have seldom been used to assess IRI in previous studies [14, 15]. In this study, the combination of 320-slice spiral CTPI and color-coded DSA values were used to evaluate IRI in a rabbit model of hindlimb, to provide guidance and a basis for the effective assessment of acute IRI in clinical practice. As of now, this is the first report comparing the use CTPI and color-coded DSA for evaluating IRI. The advantages and correlations between these two imaging techniques in the evaluation of IRI in skeletal muscle are discussed.

## Methods

### Experimental groups

Fifty New Zealand white rabbits (male, 2.0–2.5 kg) were obtained from the Huadong Xinhua Laboratory Animal Center (Guangzhou, China), and randomly assigned to the IR (*n* = 40) or control group (*n* = 10). All rabbits were raised at the Animal Laboratory Center of Jinan University, and housed with five mice per cage. After 3 h of right hindlimb ischemia, the IR group were assessed at 0, 6, 12, or 24 h (n = 10, for each time point) after reperfusion. A sham operation was performed to rabbits in the control group. For all of the IR groups, CTPI (CTPI subgroup, *n* = 5) and color-coded DSA (DSA subgroup, n = 5) were used to image the bilateral limbs of each animal, respectively.

### Creation of the IRI model in rabbits

**Fig. 1:**
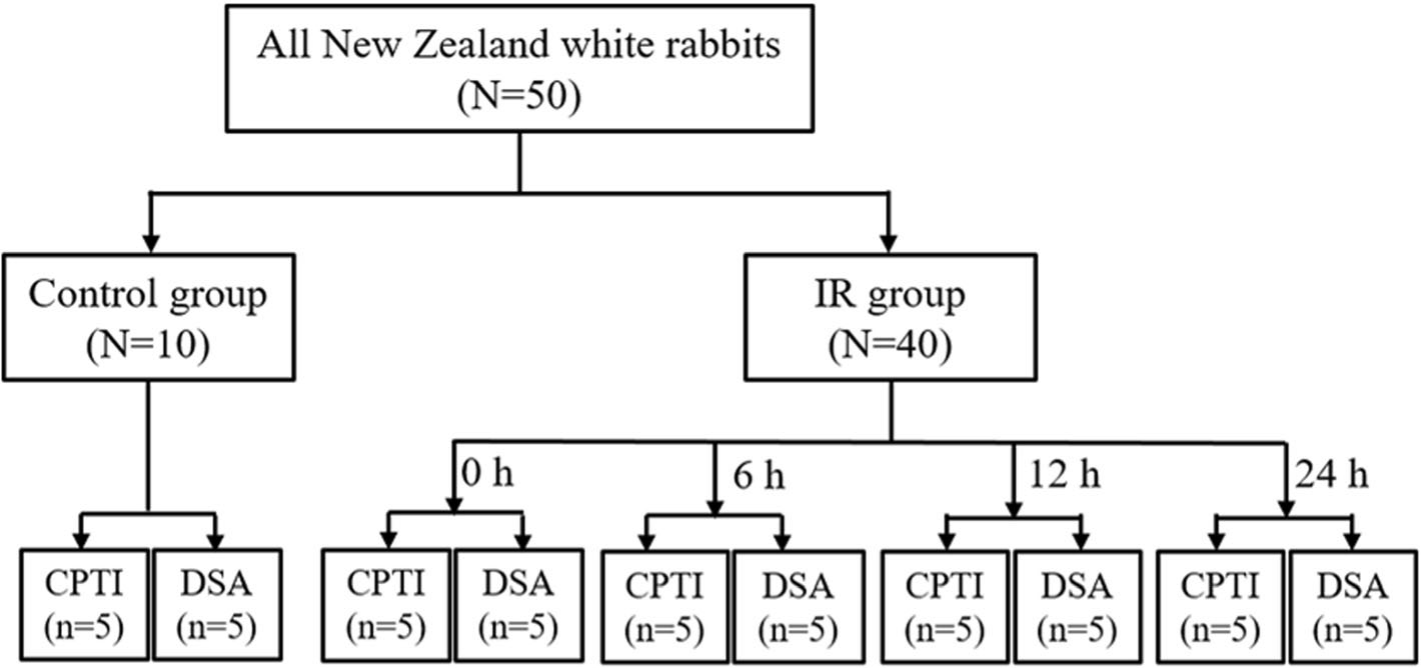
Flowchart of the present study. The time points indicate the time post-reperfusion. IR, ischemia-reperfusion; CPTI group, computed tomography perfusion imaging; DSA group, color-coded digital subtraction angiography

The rabbits were anesthetized with an intraperitoneal injection of 1% pentobarbital sodium (40 mg/kg of body weight). Using 1% pentobarbital sodium (30 mg/kg of body weight), subsequent anesthesia was maintained every 3 h through the ear-rim auricular vein injection. In the IR groups, an anterior abdominal wall incision was made after sterilization. The right iliolumbar artery, right internal iliac artery and right common iliac artery were exposed and ligated with a 3–0 silk suture (Jinhuan, Shanghai, China). The blood supply of the tail was blocked using a tight elastic band to prevent the collaterals to the hindlimb during the ischemia. After confirming successful ischemia induction, the abdomen was closed. At 3 h post-surgery, the abdomen was reopened and the ligatures were removed. The red color of vessels with a visible pulsation indicated that the reperfusion was successful. In the control group, the abdomen of rabbits was only opened and closed without ligation of the arteries. A flowchart of this study is shown as Fig. 1.

### CT scan procedures

In the CTPI subgroup of IR, the Aquilion One 320 volumetric CT scanner (Toshiba, Japan) was used to scan the bilateral hindlimbs of the experimental rabbits at 0, 6, 12, or 24 h post-reperfusion depending on the group (CT-0, CT-6, CT-12, or CT-24, respectively). After sufficient anesthesia, the rabbits were fixed at the center of the CT scanner in a supine position. The scan range included the full-length of the hindlimbs. Before scanning, 3 mL of iodixanol (32 mg/mL) was injected with a high-pressure syringe at the rate of 1 mL/s. Following the iodixanol, 8 mL of physiological saline was injected at the same rate through the ear vein. Then 3 s later, the delayed x-ray scanning was performed. The data collection time was 60.5 s which included 3 parts. In the initial 22.5 s, data was collected every 1.5 s, and the exposure time was 0.5 s. The collection was performed 12 times during this procedure. In the middle 18 s, data was collected every 2.5 s, and the exposure time was 0.5 s. The collection was performed six times during this procedure. In the last 20 s, data was collected every 4.5 s, and the exposure time was 0.5 s. The collection was performed four times during this procedure. The scanning parameters consisted of the detector arranged by 320 × 0.5 mm with a thickness of 0.5 mm, 0.5 s/ring, 80 kV, and 40 mA.

### Angiography procedures

Similar to the CTPI subgroup, rabbits in the DSA subgroup were fixed onto the operation table in supine position. After supplemental anesthesia and skin preparation, the right main carotid artery was exposed and punctured. Depending on the group (IR-0, IR-6, IR-12, or IR-24), angiography of the lower abdominal aorta was performed to assess changes in the blood flow changes of the right hindlimb using a 2.7 F microcatheter (Terumo, Japan) through the carotid artery at 0, 6, 12, or 24 h. The DSA equipment used in this study was Artis Zeego (Siemens, Erlangen, Germany). Iodixanol (320 mg/mL) was injected with a velocity of 3 mL/s through the microcatheter using a power injector. The injection pressure was 200 psi and the total dose of the contrast agent was 9 mL. The DSA acquisition frame rate was six frames per sec and each acquisition process lasted until the inferior vena cava was visualized.

### Image post-processing

The imaging data collected from CT and DSA were sent to their respective post-processing workstations, respectively (CT: Vitrea, Toshiba, Japan; DSA: Leonardo, Siemens, Germany). The CTPI and color-coded DSA images were automatically generated by the software. Based on the images, the 50-mm region of interest (ROI) was selected in the bilateral vastus lateralis muscle from all the rabbits by two practicing interventional radiologists independently. The ROI was chosen in the soft tissue, yet away from the skeleton and vessels in both the CTPI and DSA groups.

Using the post-processing workstation, perfusion parameters, including blood flow (AF), blood volume (BV), and contrast agent clearance (C) values of the right and left limb ROI (AF-R, AF-L; BV-R, BV-L; C-R, C-L, respectively) in the CTPI subgroup and the maximum contrast enhancement (peak) of the right and left limb ROI (peak-R, peak-L, respectively) in the DSA subgroup were obtained. To eliminate individual differences among the rabbits in this study, the ratios of AF, BV, C, and the peak were calculated during the post-processing (AF-R/L, BV-R/L, C-R/L and peak-R/L).

### Testing of blood samples

Prior to the CT and DSA scanning in each rabbit, 3 mL of blood was taken from the external jugular vein. After centrifugation of the blood samples, the separated serum was stored at − 20 °C. The samples were used to measure SOD, CK, LDH and MDA. CK and LDH using an automatic biochemical analyzer. In addition, MDA was measured using the thialbarbital sodium method, while SOD was measured via the xanthine oxidase method.

### Animal euthanasia

After the experiment, all rabbits were sacrificed by injecting 10 mL of air through the ear-rim auricular vein.

### Statistical methods

SPSS 16.0 (IBM, Chicago, IL, USA) was used for the statistical analysis. A single factor variance analysis was used for comparison between groups. The independent-samples *t*-test was used for comparison between data obtained from the control group and IR group. The correlation of AF-R/L, BV-R/L, C-R/L and peak-R/L between various biochemical indicators, including SOD, CK, LDH and MDA, were observed by the Pearson’s correlation analysis. The statistical data are expressed as standard error of the mean and *p*-values < 0.05 were considered significant.

## Results

### Establishment of the IRI model in rabbits

**Fig. 2:**
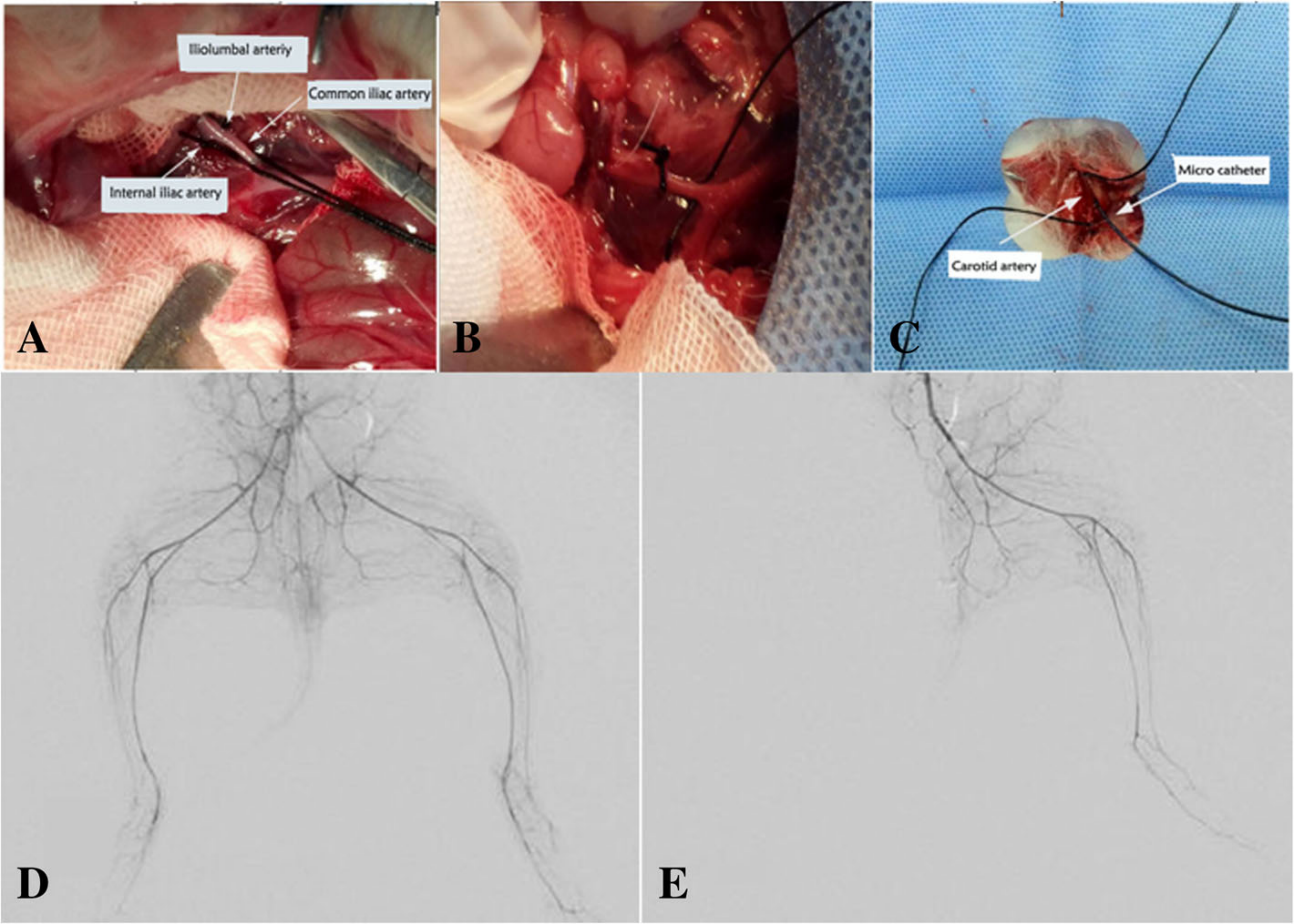
Establishment of the IRI model in rabbits was accomplished by (**a**) separating the iliolumbar artery and iliac artery, (**b**) ligating the iliolumbar artery and iliac artery, (**c**) performing a unilateral carotid artery catheterization to make the angiogram, (**d**) angiography of the bilateral hindlimbs confirmed the non-ischemia status, (**e**) angiography of the bilateral hindlimbs confirmed the ischemia status

**Fig. 3:**
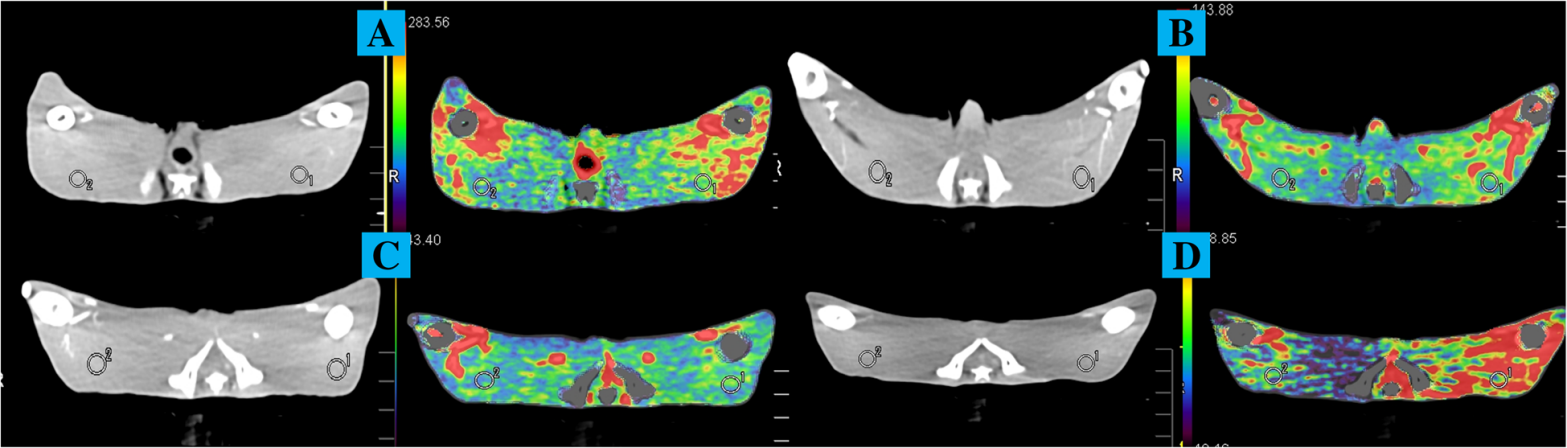
Plain CT scan and CT perfusion images in the (**a**) control group, (**b**) IR-6 h group, (**c**) IR-12 h group, and (**d**) IR-24 h group

**Fig. 4:**
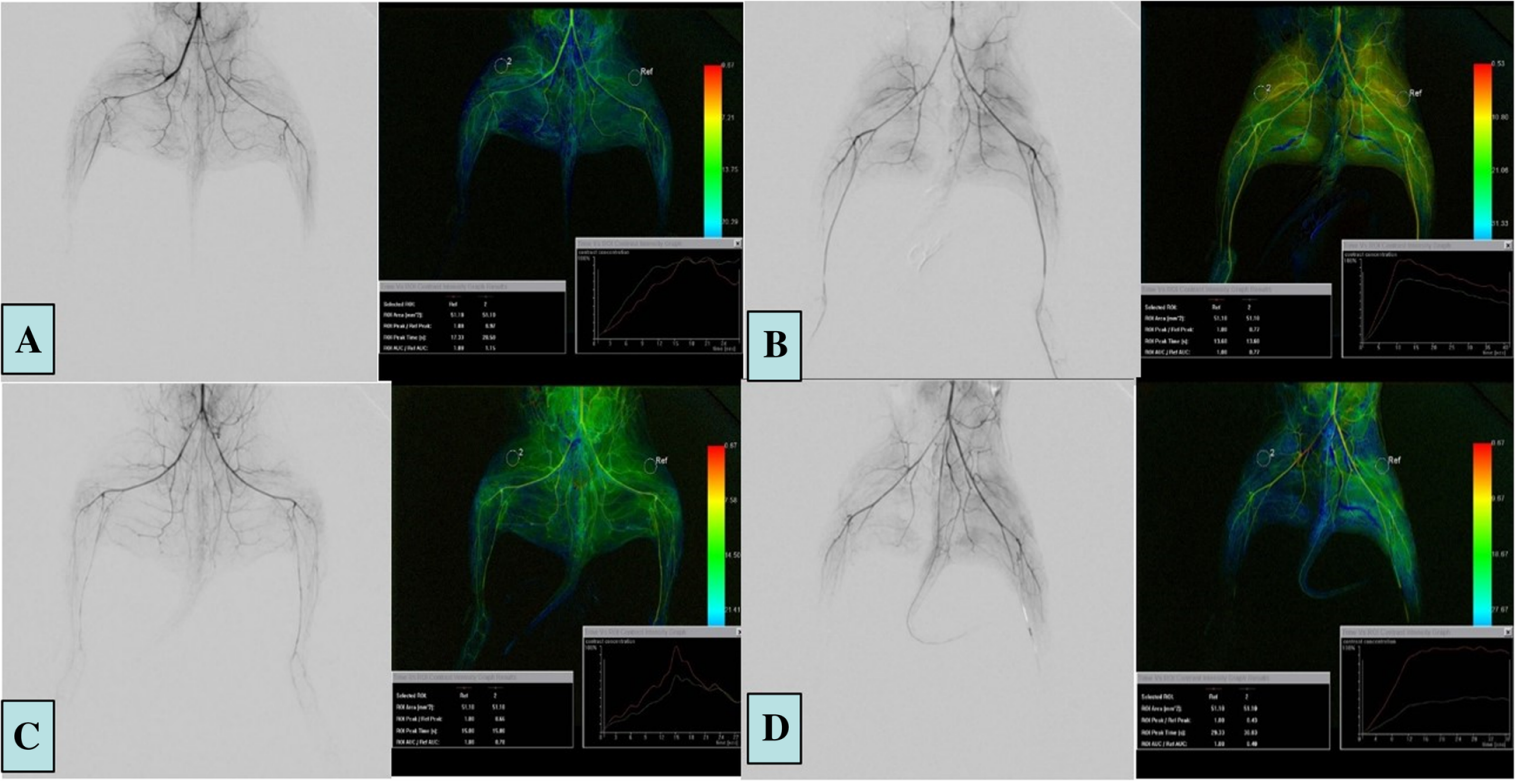
Color coded DSA post-processing images in the (**a**) control group, (**b**) IR-6 h group, (**c**) IR-12 h group, and (**d**) IR-24 h group

The surgical procedure was successful in all 45 rabbits (Fig. 2). CTPI and color-coded DSA were successfully achieved in each group (Figs. 3 and 4). There were no deaths during this study.

### CTPI and color-coded DSA parameters

The detailed CTPI and color-coded DSA parameters are shown in Tables 1 and 2. For CTPI, a significant difference existed between each experimental group and the control group in AF-R/L (*p* < 0.001). When compared with the control group, significant differences only existed in the 12 h and 24 h groups in BV-R/L and C-R/L (*p* < 0.001). Using a single factor variance analysis, significant differences in AF-R/L were detected between each group when compared with the control group (F = 6.206, *p* = 0.002). The AF-R/L value gradually decreased over time, yet this decrease was not significant between 0 h and 24 h. The BV-R/L and C-R/L from each group showed no significant differences between each other. For the color-coded DSA, there was a significant difference in Peak-R/L between the control group and all IR groups (*p* < 0.001). Extension of IRI time resulted in a gradual decrease of the Peak-R/L.

**Table 1:** CTPI parameters for each IR group and the control group (χ ± SD)

Group	AF-R/L	BV-R/L	C-R/L
Control	1.07 ± 0.08	1.03 ± 0.06	0.93 ± 0.15
IR 0 h	0.92 ± 0.14*	0.98 ± 0.18	0.83 ± 0.16
IR 6 h	0.89 ± 0.12*	0.97 ± 0.29	0.79 ± 0.08
IR 12 h	0.88 ± 0.20*	0.88 ± 0.11*	0.73 ± 0.06*
IR 24 h	0.75 ± 0.11*	0.85 ± 0.14*	0.71 ± 0.18

* *p* < 0.001 compared to the control group. *IR* ischemia-reperfusion, *AF* blood flow, *BV* blood volume, *C* contrast agent clearance

**Table 2:** Peak-R/L measurements for each IR group and the control group (χ ± SD)

Group	PEAK-R/L
Control	1.09 ± 0.04
IR 0 h	0.93 ± 0.04^*^
IR 6 h	0.80 ± 0.06^*^
IR 12 h	0.65 ± 0.03^*^
IR 24 h	0.45 ± 0.08^*^

* *p* < 0.001 compared to the control group. *IR* ischemia-reperfusion, *PEAK* maximum contrast enhancement

### Serum biomarkers for assessment of IRI

As shown in Table 3, SOD in the IR group was lower than in the control group. However, CK, LDH and MDA in the IR groups were higher than those of the sham group (*p* < 0.001). The variance analysis showed that the differences in CK and LDH among all groups were statistically significant (*p* < 0.001). CK and LDH increased gradually with the IR time. However, there were no significant differences in MDA or SOD among the groups (*p* > 0.05).

**Table 3:** Measurements of serum biochemical biomarkers from each IR group and the control group (χ ± SD)

Group	SOD (U/mL)	CK (U/L)	LDH (U/L)	MDA (nmol/mL)
Control	172.82 ± 7.06	389.75 ± 42.25	402.38 ± 26.23	1.51 ± 0.30
IR 0 h	152.14 ± 15.06^*^	688.98 ± 35.22^*^	568.65 ± 42.85^*^	2.03 ± 0.24^*^
IR 6 h	148.72 ± 15.80^*^	3668.19 ± 599.56^*^	1172.59 ± 89.86^*^	2.11 ± 0.33^*^
IR 12 h	142.24 ± 12.73^*^	5780.22 ± 421.64^*^	1410.76 ± 198.32^*^	2.38 ± 0.46^*^
IR 24 h	136.12 ± 11.40^*^	8954.13 ± 894.36^*^	2031.80 ± 265.28^*^	3.07 ± 0.60^*^

* *p* < 0.001 compared to the control group. *IR* ischemia-reperfusion, *SOD* hydrogen peroxide dismutase, *CK* creatine kinase, *LDH* lactic dehydrogenase, *MDA* malondialdehyde

### Correlation between indicators: CTPI and biochemical parameters

In the present study, AF-R/L was positively correlated with SOD (r = 0.57, *p* < 0.001) and negatively correlated with CK, LDH, and MDA (r = − 0.60, *p* < 0.001; r = − 0.44, *p* < 0.001; r = − 0.62, *P* < 0.001; respectively). LDH was negatively correlated with BV-R/L (r = − 0.45, *p* < 0.001), and no correlation existed between any of the biochemical biomarkers and C-R/L.

The Peak-R/L was positively correlated with SOD (r = 0.59, *p* < 0.001) but negatively correlated with CK, LDH and MDA (r = − 0.68, *p* < 0.001; r = − 0.71, *p* < 0.001; r = − 0.66, *p* < 0.001; respectively). The Peak-R/L was positively correlated with AF-R/L in the color-coded DSA and CTPI (r = 0.70, *p* < 0.001).

## Discussion

In the present research, the usefulness of CTPI and color-coded DSA for evaluating IRI was investigated using a hindlimb ischemia rabbit model. CTPI and DSA images were obtained from each rabbit in the CTPI and DSA subgroups at different time points (0, 6, 12, or 24 h after reperfusion). IRI-related serum biochemical biomarkers were measured and the AF-R/L, BV-R/L, C-R/L of CT perfusion and the peak-R/Ls of angiograms were determined and compared among the groups.

A series of studies have shown that CTPI is effective for assessing ISI of the myocardium, brain, and liver. These studies demonstrated that the CTPI parameters of the corresponding tissues and organs changed significantly with of reperfusion injury [16, 17]. In terms of skeletal muscle applications, CTPI can quantitatively evaluate blood flow changes during muscle tissue reperfusion in the hindlimbs of animals with acute or chronic ischemia [18]. However, there are few studies on the application of CTPI in the assessment of IRI. Our data showed that the value of AF-R/L decreased by 30% by 24 h with an obvious downward trend after IRI. Moreover, a significant correlation between AF-R/L and serum biochemical indices was observed. These findings indicate that the AF-R/L can better reflect IRI status and assessing AF-R/L in clinical work may provide additional insight into the extent of damage to skeletal muscle tissues during IRI. Hence, CPTI may be useful in the assessment of IRI.

Color-coded DSA is a kind of imaging technology implemented by post-imaging processing software. Since most studies on color-coded DSA have focused on vascular lesions, most of the literature has used the vascular lumen as the ROI. The peak time of contrast agents in the ROI was measured as a comparative parameter to evaluate hemodynamic changes [19]. While in this study, skeletal muscle IRI was the core of the research, therefore the muscle tissue was considered as the ROI. It was previously shown that IRI in muscle tissue gradually increased in the soleus muscle of rats within 24 h after 4 h of ischemia, suggesting that the most serious complications of IRI occurred within 24 h after reperfusion [5]. Hence, changes that occurred during 0–24 h post-reperfusion were investigated in the present study. Our data showed that the peak-R/Ls gradually decreased during the reperfusion procedure and was the lowest at 24 h post-reperfusion. Therefore, peak-R/L can reflect the severity of reperfusion injury to a certain extent, indicating color-coded DSA can be used to evaluate IRI.

Laboratory measurements of SOD, CK, LDH and MDA can also be used to objectively evaluate the extent and severity of IRI [6, 20–22]. This study also investigated the relationship between these biochemical parameters and IRI. The results showed serum levels of CK and LDH were higher in the IRI when compared with the control group. Moreover, these parameters positively correlated with the duration of reperfusion, which suggests that rabbit limbs were damaged by the reperfusion and the damage gradually increased over time. Similarly, MDA gradually increased with the duration of reperfusion, while SOD decreased over time after reperfusion. SOD and MDA indirectly reflected the content of free oxygen radicals, and the increase of MDA and decrease of SOD represents an increased production of free oxygen radicals [23]. Thus, our data indicate that oxidative injury of tissues gradually increases with the duration of IRI.

Our findings suggest that both AF-R/L from CTPI and peak-R/L from color-coded DSA can be used to objectively access IRI. In clinical work, CTPI and color-coded DSA have respective advantages in evaluating IRI. CTPI is a non-invasive approach that is more widely accepted by patients and physicians. Also, CTPI data are derived from sliced images, which increases its accuracy, especially in terms of AF-R/L. However, the patient is exposed to ionizing radiation via the CT scan and contrast agent [10]. Despite the invasiveness of DSA, it has been considered the gold standard for detecting vascular diseases [24]. Compared with CT, DSA requires less time and can be completed during an operation, which can aid surgeons in the identification of disease severity. Furthermore, color-coded DSA can be generated from a conventional DSA sequence with no additional x-ray dose or contrast medium [14]. Our findings suggest that both AF-R/L from CTPI and peak-R/L from color-coded DSA were indicators of IRI, and revealed that IRI worsened with increased time to reperfusion. Moreover, they were positively correlated and also related with some serum biomarkers of IRI. Thus, CPTI and color-coded DSA can be chosen according to the actual status of patients in clinical application.

However, there are several limitations to our study. The imaging changes observed during reperfusion cannot be distinguished from those changes caused by the ischemic injury. Therefore, further comparative studies assessing these two forms of injury, including reperfusion and ischemia-induced injury, should be conducted in the future.

## Conclusions

In conclusion, the combination of 320-slice spiral CT perfusion analysis and color-coded DSA allows for the dynamic detection and assessment of disease occurrence and severity in rabbits with skeletal muscle IRI.

## Data Availability

The datasets used and/or analyzed during the current study are available from the corresponding author on reasonable request.

## Abbreviations

CK: Creatine kinase
CTPI: Computed tomography perfusion imaging
DSA: Digital subtraction angiography
IR: Ischemia-reperfusion
IRI: Ischemia-reperfusion injury
LDH: Lactic dehydrogenase
MDA: Malondialdehyde
MRI: Magnetic resonance imaging
SOD: Superoxide dismutase
